# Subcutaneous *Aspergillus* nodule with cutaneous *Enterococcus* infection

**DOI:** 10.1093/omcr/omab082

**Published:** 2021-09-13

**Authors:** Makoto Kondo, Keiichi Yamanaka

**Affiliations:** Department of Dermatology, Mie University, Graduate School of Medicine, Tsu, Mie 514-8507, Japan

## Abstract

An 87-year-old woman presented with a subcutaneous nodule with overlying black and yellow scales on the surface located on the left forearm. *Enterococcus faecalis* grew up in bacterial culture using specimen from skin surface scale. And *Aspergillus fumigatus* was detected in the subcutaneous tissue culture. When suspecting a deep infection, not only the surface part but also the deeper part must be cultured, because we may mislead about the identity of the infectious organism only bacterial culture form skin surface.

## CLINICAL IMAGES

**Figure 1:**
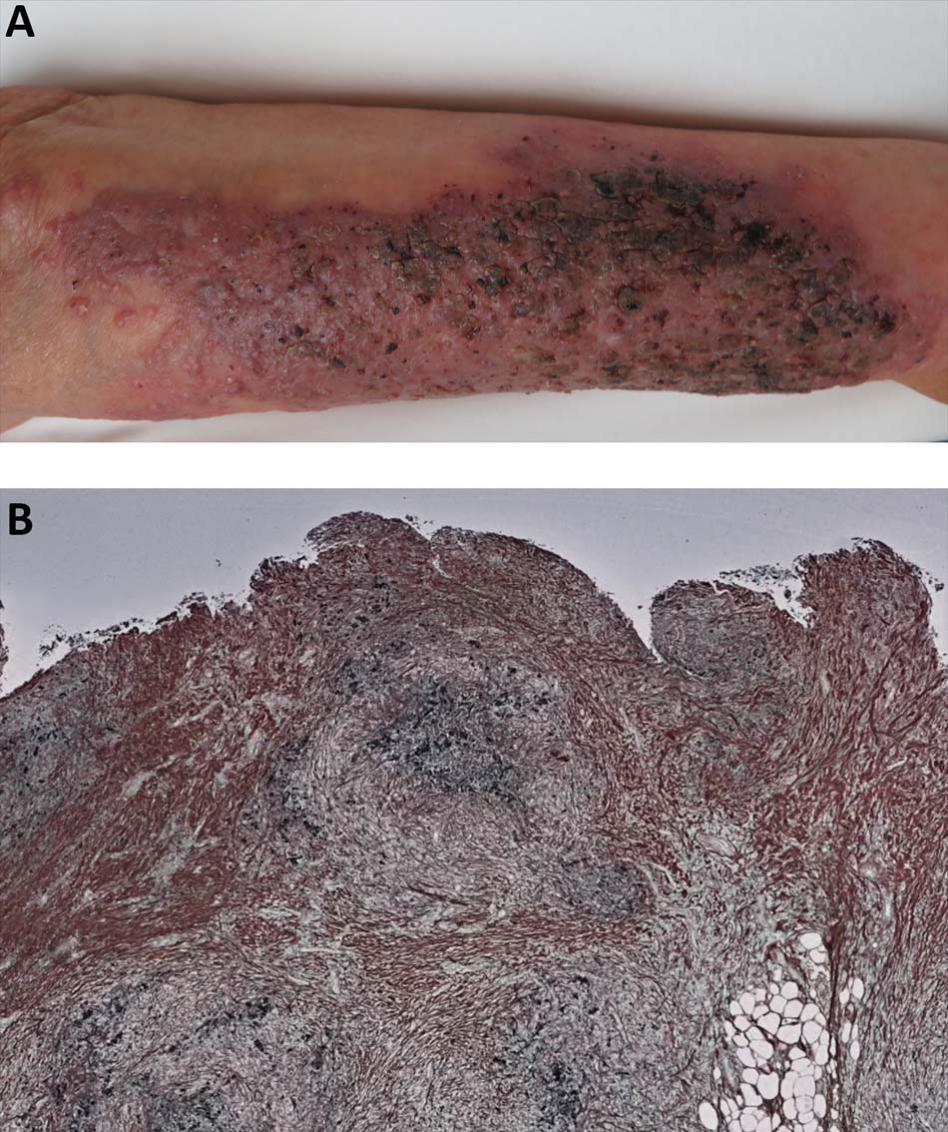
(a) Left arm with large erythematous plaque with crusting, yellow and black scales on subcutaneous nodules; (b) Subcutaneous abscess positive for Grocott staining.

An 87-year-old woman with a history of idiopathic interstitial pneumonia and receiving an immune suppressant drug (17.5 mg/day of prednisolone and 4 mg/day of tacrolimus) presented with a subcutaneous nodule with overlying black and yellow scales on the surface located on the left forearm (Fig. 1). The burning sensation was accompanied and erythema was noticed around the nodule. CRP was 0.56 (normal range < 0.30 pg/ml) and WBC count was 9060/μl (neutro 6140/μl and monocyte 670/μl). The other hand, β-d glucan showed high levels of 230 pg/ml (normal range < 20 pg/ml). *Enterococcus faecalis* grew up in bacterial culture using specimen from skin surface scale. Skin biopsy revealed a subcutaneous abscess positive for Grocott staining (Fig. 1b), and *Aspergillus fumigatus* was detected in the subcutaneous tissue culture. Whole body computed tomography showed no sign of any other fungal infection. However, the genome sequencing data for the fungal Internal Transcribed Spacer and bacterial 16srRNA using the Next Generation Sequencer revealed the disappearance of bacterial diversity from the superficial scale, with *E. faecalis* being the dominant species. No fungus was detected. The microbiome in healthy skin is usually abundant of several species [1]. The nodule and scale disappeared gradually following therapies for both *Aspergillus* and *Enterococcus* (100 mg/day of voriconazole, 80 mg of trimethoprim and 40 mg of sulfamethoxazole). When suspecting a deep infection, not only the surface part but also the deeper part must be cultured, because we may mislead about the identity of the infectious organism only bacterial culture form skin surface.

## CONFLICT OF INTTEREST STATEMENT

The authors have declared that no competing interests exist.

## FUNDING

The authors did not receive any financial support for the present study.

## ETHICAL APPROVAL

This case report was conducted according to the principles of the Declaration of Helsinki.

## CONSENT

Written informed consent was obtained from the patient.

## GUARANTOR

Makoto Kondo.
